# Efficacy and safety of fixed-dose combinations of aclidinium bromide/formoterol fumarate: the 24-week, randomized, placebo-controlled AUGMENT COPD study

**DOI:** 10.1186/s12931-014-0123-0

**Published:** 2014-10-14

**Authors:** Anthony D D’Urzo, Stephen I Rennard, Edward M Kerwin, Victor Mergel, Anne R Leselbaum, Cynthia F Caracta, on behalf of the AUGMENT COPD study investigators

**Affiliations:** University of Toronto, Toronto, Canada; University of Nebraska Medical Center, Omaha, NE USA; Clinical Research Institute, Medford, OR USA; Forest Research Institute, Jersey City, NJ USA; R&D Centre, Almirall, Barcelona, Spain

**Keywords:** Aclidinium, Formoterol, COPD, Lung function, Dyspnea, Health status

## Abstract

**Background:**

Combining two long-acting bronchodilators with complementary mechanisms of action may provide treatment benefits to patients with chronic obstructive pulmonary disease (COPD) that are greater than those derived from either treatment alone. The efficacy and safety of a fixed-dose combination (FDC) of aclidinium bromide, a long-acting muscarinic antagonist, and formoterol fumarate, a long-acting β_2_-agonist, in patients with moderate to severe COPD are presented.

**Methods:**

In this 24-week double-blind study, 1692 patients with stable COPD were equally randomized to twice-daily treatment with FDC aclidinium 400 μg/formoterol 12 μg (ACL400/FOR12 FDC), FDC aclidinium 400 μg/formoterol 6 μg (ACL400/FOR6 FDC), aclidinium 400 μg, formoterol 12 μg, or placebo administered by a multidose dry powder inhaler (Genuair^®^/Pressair^®^)*. Coprimary endpoints were change from baseline to week 24 in 1-hour morning postdose FEV_1_ (FDCs versus aclidinium) and change from baseline to week 24 in morning predose (trough) FEV_1_ (FDCs versus formoterol). Secondary endpoints were change from baseline in St. George’s Respiratory Questionnaire (SGRQ) total score and improvement in Transition Dyspnea Index (TDI) focal score at week 24. Safety and tolerability were also assessed.

**Results:**

At study end, improvements from baseline in 1-hour postdose FEV_1_ were significantly greater in patients treated with ACL400/FOR12 FDC or ACL400/FOR6 FDC compared with aclidinium (108 mL and 87 mL, respectively; p < 0.0001). Improvements in trough FEV_1_ were significantly greater in patients treated with ACL400/FOR12 FDC versus formoterol (45 mL; p = 0.0102), a numerical improvement of 26 mL in trough FEV_1_ over formoterol was observed with ACL400/FOR6 FDC. Significant improvements in both SGRQ total and TDI focal scores were observed in the ACL400/FOR12 FDC group at study end (p < 0.0001), with differences over placebo exceeding the minimal clinically important difference of ≥4 points and ≥1 unit, respectively. All treatments were well tolerated, with safety profiles of the FDCs similar to those of the monotherapies.

**Conclusions:**

Treatment with twice-daily aclidinium 400 μg/formoterol 12 μg FDC provided rapid and sustained bronchodilation that was greater than either monotherapy; clinically significant improvements in dyspnea and health status were evident compared with placebo. Aclidinium/formoterol FDC may be an effective and well tolerated new treatment option for patients with COPD.

**Trial registration:**

Clinicaltrials.gov NCT01437397.

*Registered trademarks of Almirall S.A., Barcelona, Spain; for use within the US as Pressair^®^ and Genuair^®^ within all other licensed territories.

**Electronic supplementary material:**

The online version of this article (doi:10.1186/s12931-014-0123-0) contains supplementary material, which is available to authorized users.

## Background

In patients with chronic obstructive pulmonary disease (COPD), combining bronchodilators with complementary mechanisms of action has the potential to increase lung function and improve symptom management compared to treatment with a single agent [1]. Inhaled long-acting muscarinic antagonists (LAMAs) and long-acting β_2_-agonists (LABAs) are widely used as maintenance treatment in COPD. LAMAs indirectly reduce bronchoconstriction by inhibiting acetylcholine signaling via muscarinic receptors on airway smooth muscle, while LABAs directly stimulate β_2_-adrenoceptors that lead to smooth muscle relaxation. Though the mechanisms of action of these two classes of bronchodilators differ, LAMA/LABA combinations have been a successful treatment option for patients with COPD, improving both spirometric values and health-related quality of life [2-4].

Current treatment guidelines recommend LAMA/LABA combination therapy for COPD patients uncontrolled by bronchodilator monotherapy [1]. Studies investigating the free combination of LAMA and LABA therapies (via two separate inhalers) in patients with COPD have shown improved bronchodilation and reduced rescue medication use compared with monotherapy [2,3,5,6]. Treatment with either aclidinium bromide (a LAMA) 400 μg twice-daily (BID) or formoterol fumarate (a LABA) 12 μg twice daily improves lung function and reduces COPD symptoms while being well tolerated [7-12]. As treatment with aclidinium also has been shown to improve health status, exercise endurance, and nighttime symptoms in patients with COPD [11,13], a fixed-dose combination (FDC) comprising aclidinium bromide/formoterol fumarate (Genuair^®^/Pressair^®^*, approved for delivery of aclidinium monotherapy) may improve lung function, health status, and COPD symptoms while reducing the potential risk for side effects that often occur from increasing doses of monotherapy treatments [1].

The efficacy and safety of twice-daily aclidinium/formoterol FDC in patients with moderate to severe COPD were assessed in a 24-week phase 3, randomized, double-blind study (AUGMENT COPD, **A**clidinium/formoterol F**U**marate Combination for Investi**G**ative use in the Treat**MENT** of Moderate to Severe COPD), the results of which are presented here.

## Methods

### Study design

**Figure 1:**
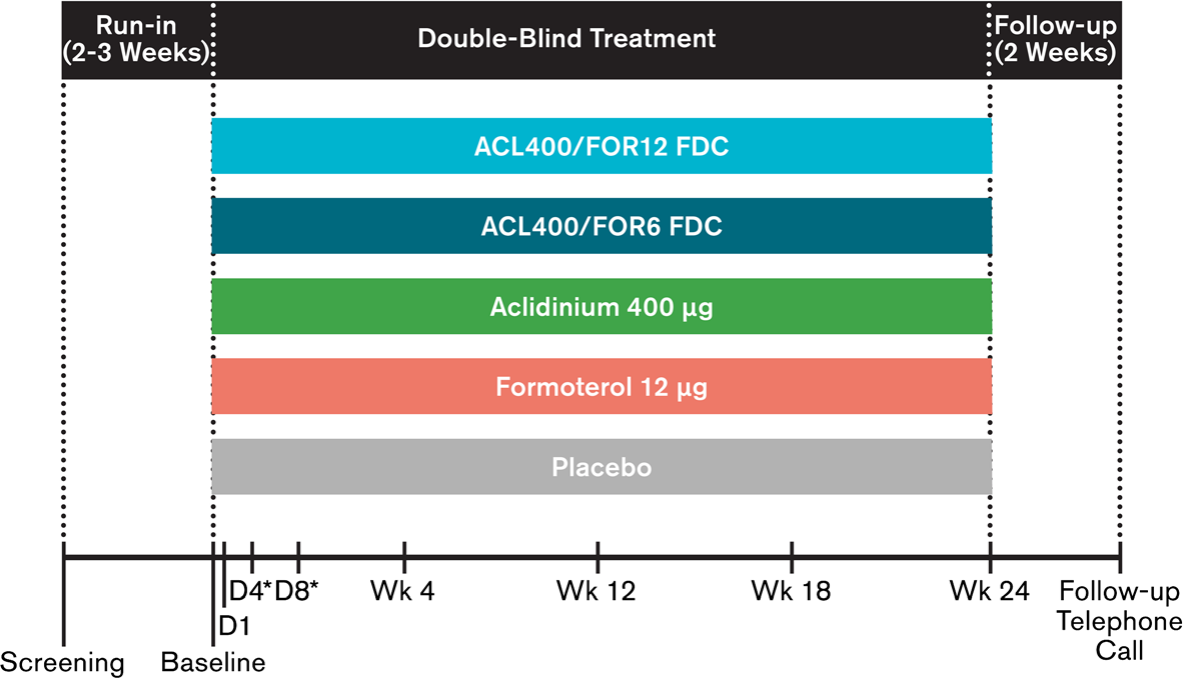
**Study design.** ACL400/FOR12 FDC, fixed-dose combination of aclidinium 400 μg and formoterol 12 μg; ACL400/FOR6 FDC, fixed-dose combination of aclidinium 400 μg and formoterol 6 μg; D, day; Wk, week.

This phase 3, randomized, double-blind study in patients with moderate to severe COPD was conducted in 222 centers throughout North America, Australia, and New Zealand (NCT01437397) in accordance with the International Conference on Harmonization/Good Clinical Practice guidelines and the Declaration of Helsinki. The protocol was approved by the Institutional Review Board at each study center, and all patients gave written informed consent before participating in any study procedures. The study comprised a 2- to 3-week run-in period prior to a 24-week double-blind treatment period (Figure 1). Patients were equally randomized to twice-daily aclidinium/formoterol FDC 400/12 μg, aclidinium/formoterol FDC 400/6 μg, aclidinium 400 μg, formoterol 12 μg, or placebo, administered via a multidose, dry-powder inhaler (Genuair^®^/Pressair^®^)*. The first patient/first visit was October 4, 2011 and the last patient completed February 6, 2013.

### Patients

Patients aged ≥40 years were eligible if they were current or former smokers (≥10 pack-years) and diagnosed with stable, moderate to severe expiratory airflow obstruction according to GOLD guidelines (postbronchodilator forced expiratory volume in 1 second [FEV_1_]/forced vital capacity [FVC] <70% and FEV_1_ ≥30% and <80% predicted) [1]. Main exclusion criteria were: COPD exacerbation or respiratory tract infection ≤6 weeks (≤3 months if hospitalized for exacerbation) before screening; clinically significant respiratory conditions (including asthma); clinically significant cardiovascular (CV) conditions including myocardial infarction (MI) within the previous 6 months; unstable angina; and, unstable arrhythmia that required changes in pharmacological therapy or other intervention within the previous 6 months. Use of long-acting bronchodilators other than investigative treatment was not permitted. Other COPD medications, such as theophylline, inhaled corticosteroids (ICS), oral or parenteral corticosteroids (≤10 mg/day or 20 mg every other day of prednisone) were allowed if treatment was stable ≥4 weeks prior to screening. Use of albuterol/salbutamol as rescue medication was permitted.

### Outcomes and assessments

Coprimary efficacy parameters, assessed by standardized spirometric measurements of lung function [14], were change from baseline to week 24 in 1-hour morning postdose FEV_1_ (each FDC versus aclidinium, contribution of formoterol) and change from baseline to week 24 in morning predose (trough) FEV_1_ (each FDC versus formoterol, contribution of aclidinium). Secondary efficacy parameters were change from baseline in St. George’s Respiratory Questionnaire (SGRQ) total score at week 24 (each FDC versus placebo) and improvement in Transition Dyspnea Index (TDI) focal score at week 24 (each FDC versus placebo).

Additional treatment comparisons for each coprimary parameter included: each aclidinium/formoterol FDC dose versus each monotherapy; each active treatment versus placebo; and aclidinium/formoterol FDC 400/12 μg versus aclidinium/formoterol FDC 400/6 μg. Additional efficacy parameters included: change from baseline in peak FEV_1_ at all visits; 12-hour spirometry measurements (in a subset of the intention-to-treat [ITT] population) based on change from baseline in FEV_1_ at all study visits; SGRQ and Baseline/Transition Dyspnea Index (BDI/TDI) at all study visits except week 24; rescue medication use; onset of action of bronchodilation; and, daily COPD symptoms assessed by the Exacerbations of Chronic Pulmonary Disease Tool (EXACT)-Respiratory Symptoms (E-RS) questionnaire [15]. A Nighttime Symptoms of COPD Instrument (NiSCI) [16,17] and an Early Morning Symptoms of COPD Instrument (EMSCI) [18]—newly developed patient reported outcome measures undergoing empirical testing—were completed twice daily by patients using the electronic diary.

Safety was assessed through reporting of adverse events (AEs), clinical laboratory tests, vital signs, electrocardiograms (ECGs), and 24-hour 12-lead Holter monitoring. Major adverse cardiovascular events (MACE) were defined as the composite of CV deaths, nonfatal MIs, and nonfatal strokes. MACE were evaluated and classified by an adjudication committee of independent cardiologists who were not participating in the study and were blinded to treatment. To identify all MACE, a list of all AEs that were reported in randomized patients based on standard medical dictionary for regulatory activities (MedDRA) queries of cardiac disorders and cerebrovascular disorders was used.

Assessments for all efficacy and safety outcomes occurred at various timepoints throughout the study (Additional file 1: Table S1).

### Statistical analysis

Statistical analyses were performed using SAS^®^ version 9.2. All efficacy analyses with the exception of E-RS were based on the ITT population, defined as all randomized patients who took ≥1 dose of study medication and had a baseline and at least one postbaseline FEV_1_ assessment. E-RS data were analyzed for the ITT-Exacerbation Population, which included all patients in the randomized population who took at least 1 dose of double-blind investigational product. Change from baseline in bronchodilation outcomes were analyzed by mixed model for repeated measures (MMRM) with treatment group, sex, smoking status, visit, and treatment-group-by-visit interaction as fixed-effect factors and corresponding baseline values and age as covariates, and pre- and postbronchodilator FEV_1_ as a covariate for FEV_1_ outcomes. A sample size of 1550 (310 per randomized group) was estimated to provide at least 90% power to detect a statistically significant treatment difference of 100 mL (standard deviation of 280 mL) between each FDC dose versus aclidinium monotherapy in change from baseline at 1-hour morning postdose FEV_1_ at week 24, and 65 mL (standard deviation of 240 mL) between each FDC dose versus formoterol monotherapy in the change from baseline in morning predose (trough) FEV_1_ at week 24. To control for family-wise type 1 error rate at the 2-sided 5% significance level for the primary and secondary efficacy endpoints, a prespecified multiple comparison strategy was conducted.

The onset of action of bronchodilation in FEV_1_ (an additional endpoint defined as a >15% increase from baseline in FEV_1_) from 5-minutes to 3-hours postdose on day 1 was evaluated using a logistic regression model with treatment groups, sex, and smoking status as fixed-effect factors and pre- and postbronchodilator FEV_1_ at screening, age, and baseline FEV_1_ as covariates. Odds ratios were estimated for each treatment group versus placebo. Change from baseline in TDI and SGRQ were analyzed using a MMRM as described for bronchodilation outcomes. A logistic random-effect model was used to analyze the number and percentage of patients who achieved a clinically meaningful improvement from baseline in SGRQ total score (decrease of ≥4 units) [19] or in TDI focal score (increase of ≥1 unit). Use of rescue medication was analyzed using averages of the daily diary values over the time periods between visits and were based on the change from baseline values. Daily COPD symptoms were analyzed by means of an MMRM adjusted for baseline, treatment, visit, sex, age, smoking status, and treatment-by-visit interaction. Safety results, summarized descriptively, were based on the safety population, defined as all randomized patients who took ≥1 dose of study medication.

## Results

### Patient disposition and baseline characteristics

**Figure 2:**
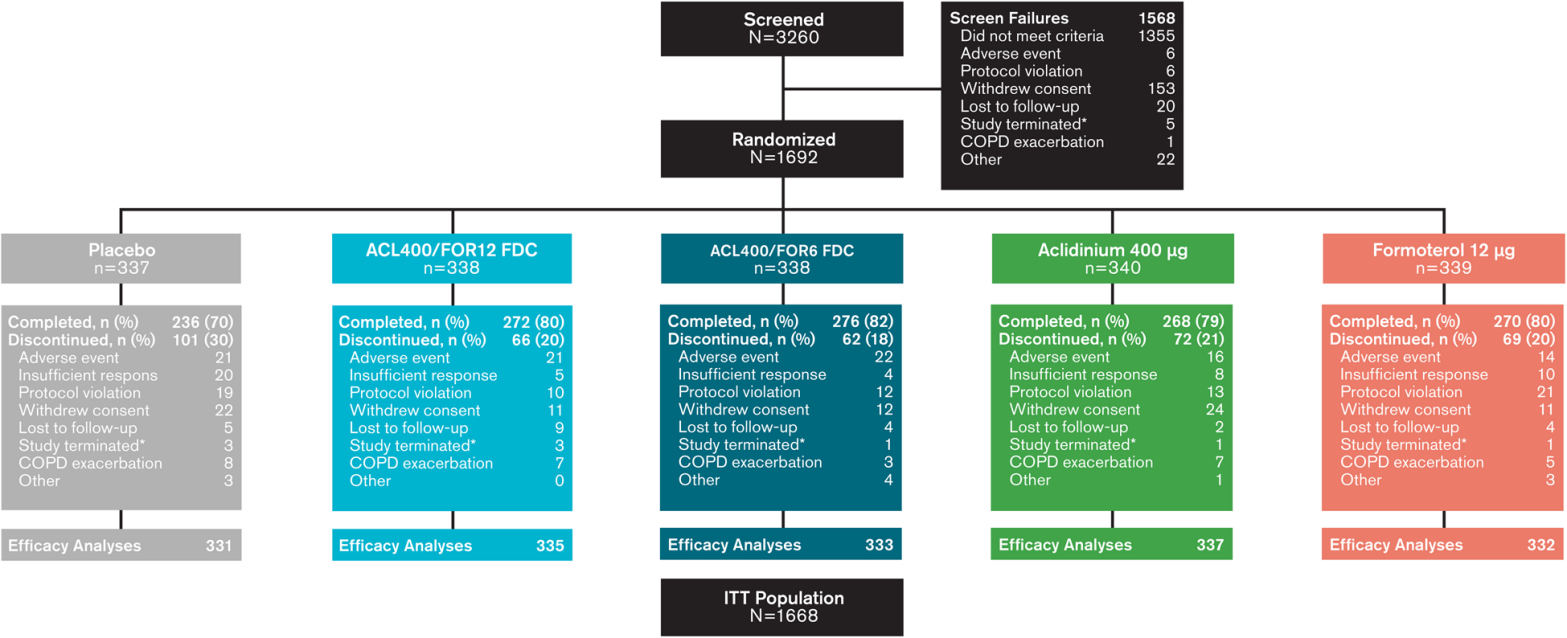
**Patient disposition.** ^*^Study terminated by Sponsor; ACL400/FOR12 FDC, fixed-dose combination of aclidinium 400 μg and formoterol 12 μg; ACL400/FOR12 FDC, fixed-dose combination of aclidinium 400 μg and formoterol 6 μg; COPD, chronic obstructive pulmonary disease; ITT, intention-to-treat.

A total of 1692 patients were randomized (Figure 2). Completion rates were highest with aclidinium/formoterol FDC 400/12 μg (80.5%) and aclidinium/formoterol FDC 400/6 μg (81.7%) and lowest with placebo (70.0%). Study discontinuations among randomized groups were most frequently due to AEs, protocol violation, and withdrawal of consent. A total of 5.9% of patients in the placebo group discontinued due to insufficient therapeutic response. Among the active treatment groups, discontinuations due to insufficient response were 2.9% for formoterol, 2.4% for aclidinium, and 1.5% and 1.2% for the aclidinium/formoterol FDC 400/12 μg and 400/6 μg groups.

Patient demographics and baseline characteristics were similar across groups (Table 1). The average patient age was 63.9 years, 53.1% were male, and 93.2% were white. Average postbronchodilator FEV_1_% predicted at screening was 53.5% and baseline FEV_1_ was 1.37 L. There were no notable differences in demographics between the ITT and safety populations.

**Table 1:** **Demographic and baseline characteristics** ^**a**^

**Characteristics, mean (SD)** ^**b**^	**PBO (n = 332)**	**ACL400/FOR12 FDC (n = 335)**	**ACL400/FOR6 FDC (n = 333)**	**ACL 400 (n = 337)**	**FOR 12 (n = 332)**
Age, years	63.5 (8.9)	64.2 (8.9)	63.9 (9.2)	64.4 (8.7)	63.7 (8.7)
Male, n (%)	175 (52.7)	168 (50.1)	187 (56.2)	188 (55.8)	169 (50.9)
White, n (%)	317 (95.5)	305 (91.0)	309 (92.8)	314 (93.2)	311 (93.7)
Current smoker, n (%)	169 (50.9)	173 (51.6)	176 (52.9)	171 (50.7)	171 (51.5)
Smoking history, pack-years	53.3 (28.5)	53.3 (27.2)	52.1 (25.8)	52.0 (26.1)	52.5 (23.7)
Prebronchodilator FEV_1_, L	1.35 (0.54)	1.34 (0.53)	1.40 (0.54)	1.34 (0.53)	1.37 (0.52)
Postbronchodilator FEV_1_, % of predicted	52.6 (13.3)	53.2 (13.4)	54.7 (12.9)	53.0 (13.3)	53.9 (13.1)
Bronchial reversibility, %	18.4 (15.2)	17.2 (14.6)	17.7 (15.0)	19.1 (16.5)	17.3 (14.7)
COPD severity, n (%)^c^					
Moderate	177 (53.3)	189 (56.4)	203 (61.0)	184 (54.6)	197 (59.3)
Severe	150 (45.2)	142 (42.4)	127 (38.1)	147 (43.6)	131 (39.5)
Baseline efficacy variables^d^					
SGRQ total score	45.3 (17.9)	47.6 (16.9)	46.2 (17.9)	45.2 (17.8)	45.8 (17.9)
BDI focal score	6.4 (2.4)	6.2 (2.1)	6.5 (2.2)	6.5 (2.3)	6.3 (2.3)
Rescue medication use, puffs/day	4.2 (3.9)	4.5 (3.7)	4.0 (3.3)	4.0 (3.4)	4.3 (3.7)
EXACT-RS score	11.03 (5.84)	11.89 (6.51)	11.46 (6.25)	11.27 (6.33)	11.40 (6.57)
NiSCI score	0.95 (0.63)	1.10 (0.70)	0.99 (0.69)	1.00 (0.70)	0.99 (0.71)
EMSCI score	1.07 (0.58)	1.19 (0.63)	1.15 (0.64)	1.13 (0.65)	1.13 (0.66)

^a^For the safety population, unless indicated otherwise; ^b^All results reported as mean values with standard deviations, unless indicated otherwise; ^c^COPD severity based on GOLD 2011 update guidelines [32]. A small (<2%) portion of the population (not shown here) were diagnosed as having mild or very severe COPD at baseline; ^d^For the intention-to-treat population: PBO, n = 331; ACL400/FOR12 FDC, n = 335; ACL400/FOR6 FDC, n = 333; ACL 400, n = 337; FOR 12, n = 332; Total, N = 1669.

ACL 400, aclidinium 400 μg; ACL400/FOR12 FDC, fixed-dose combination of aclidinium 400 μg and formoterol 12 μg; ACL400/FOR6 FDC, fixed-dose combination of aclidinium 400 μg and formoterol 6 μg; BDI, Baseline Dyspnea Index; COPD, chronic obstructive pulmonary disease; FEV_1_, forced expiratory volume in 1 second; FOR 12, formoterol 12 μg; SGRQ, St. George’s Respiratory Questionnaire; PBO, placebo; SD, standard deviation.

### Efficacy

#### 1-hour postdose FEV_1_

**Figure 3:**
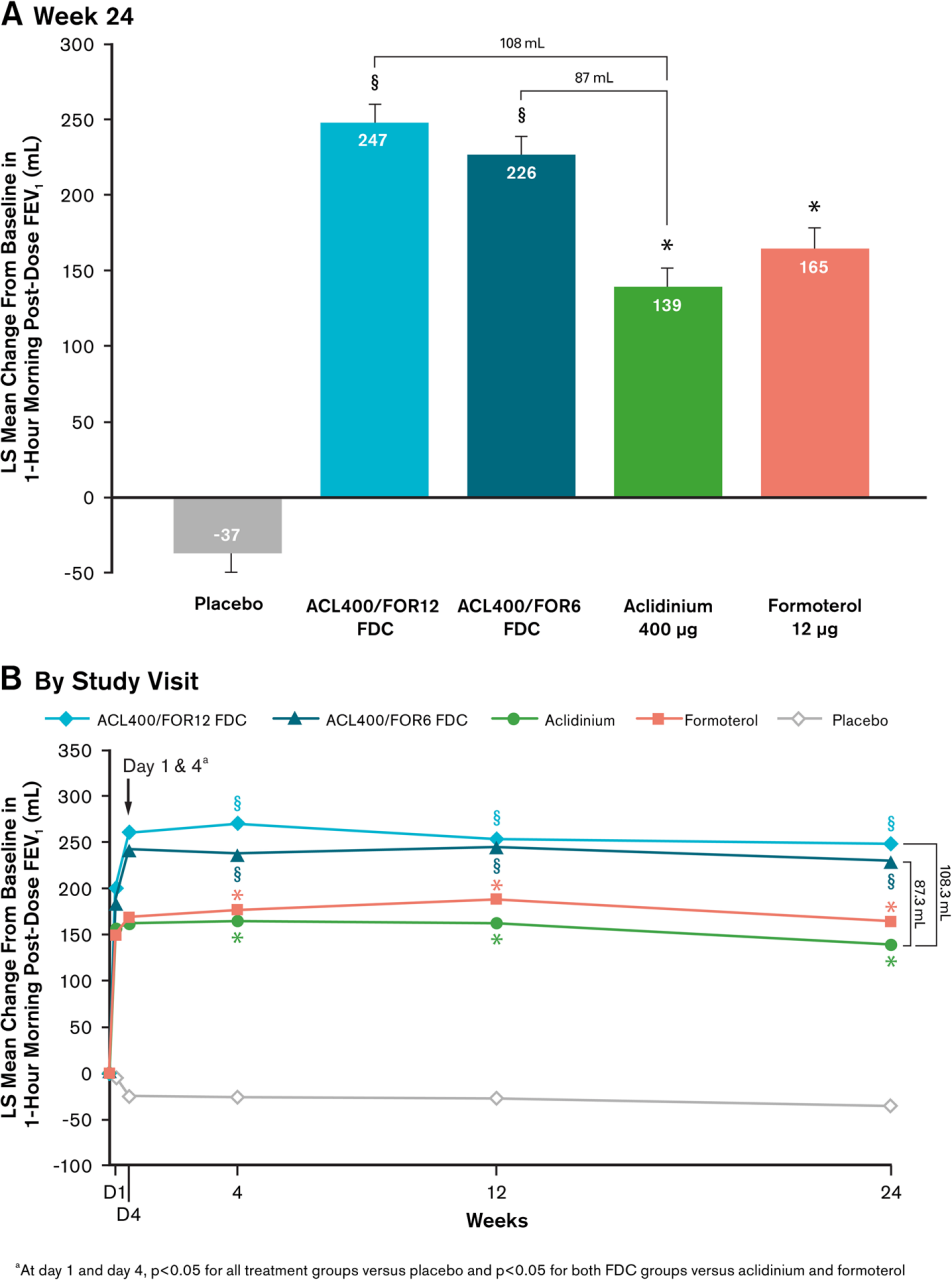
**Mean changes from baseline in 1-hour morning postdose FEV**_**1**_ **(A) at week 24 (coprimary endpoint) and (B) over time across duration of study.** Analyses were based on a mixed-model for repeated measures. All active treatment groups were significant versus placebo (p < 0.0001) at all study visits. ^*^p < 0.05 versus placebo; ^§^p < 0.05 versus aclidinium, formoterol, and placebo. ACL400/FOR12 FDC, fixed-dose combination of aclidinium 400 μg and formoterol 12 μg; ACL400/FOR6 FDC, fixed-dose combination of aclidinium 400 μg and formoterol 6 μg; D1, day 1; D4, day 4; FEV_1_, forced expiratory volume in 1 second; LS, least squares.

Treatment with both aclidinium/formoterol FDC doses resulted in clinically meaningful and significant improvements in lung function, measured by the change from baseline to week 24 in 1-hour postdose FEV_1_ versus aclidinium monotherapy (coprimary endpoint), with least squares (LS) mean treatment differences of 108 mL (aclidinium/formoterol FDC 400/12 μg) and 87 mL (aclidinium/formoterol FDC 400/6 μg) (Figure 3A, p < 0.0001). At all timepoints from the first dose, treatment with either aclidinium/formoterol FDC 400/12 μg or aclidinium/formoterol FDC 400/6 μg resulted in significant improvements from baseline in 1-hour postdose FEV_1_ compared with aclidinium, formoterol, and placebo (Figure 3B; p < 0.01 for all). At all timepoints, both monotherapies resulted in significantly greater improvements from baseline compared with placebo (p < 0.0001 for all). A numerically greater change from baseline in 1-hour postdose FEV_1_ was evident for aclidinium/formoterol FDC 400/12 μg compared with aclidinium/formoterol FDC 400/6 μg at all timepoints, including 24 weeks, though the improvements did not reach statistical significance except at week 4 (p < 0.05).

#### Trough FEV_1_

**Figure 4:**
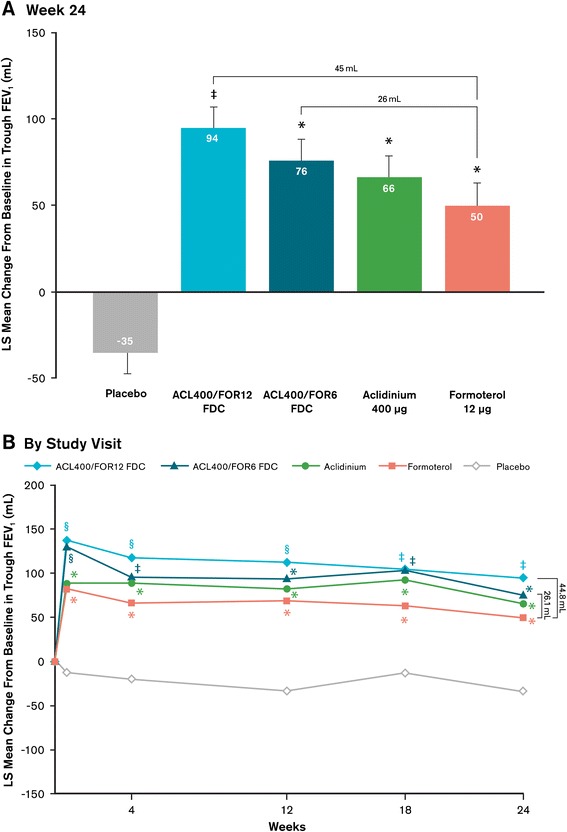
**Mean changes from baseline in morning predose (trough) FEV**_**1**_ **(A) at week 24 (coprimary endpoint) and (B) over time across duration of study.** Analyses were based on a mixed-model for repeated measures. ^*^p < 0.05 versus placebo; ^‡^p < 0.05 versus formoterol and placebo; ^§^p < 0.05 versus aclidinium, formoterol, and placebo. ACL400/FOR12 FDC, fixed-dose combination of aclidinium 400 μg and formoterol 12 μg; ACL400/FOR6 FDC, fixed-dose combination of aclidinium 400 μg and formoterol 6 μg; FEV_1_, forced expiratory volume in 1 second; LS, least squares.

Treatment with aclidinium/formoterol FDC 400/12 μg significantly improved morning predose (trough) FEV_1_ from baseline compared with formoterol at week 24 (coprimary endpoint), with an LS mean difference of 45 mL (Figure 4A, p = 0.01); treatment with aclidinium/formoterol FDC 400/6 μg resulted in a numerically greater improvement from baseline versus formoterol at study end (LS mean treatment difference, 26 mL; p = 0.133). Significant improvements from baseline in trough FEV_1_ were observed with aclidinium/formoterol FDC 400/12 μg compared with either monotherapy at all timepoints (p < 0.05 for all) except week 18 and 24 versus aclidinium (Figure 4B). At various timepoints throughout the study, both FDCs improved trough FEV_1_ from baseline versus one or both monotherapies. Compared with placebo, both aclidinium/formoterol FDCs and the monotherapies significantly improved trough FEV_1_ from baseline at all timepoints (p < 0.0001 for all). At all timepoints throughout the study, numerically greater improvements from baseline were observed in trough FEV_1_ for the aclidinium/formoterol FDC 400/12 μg versus aclidinium/formoterol FDC 400/6 μg dose.

#### Onset of bronchodilation

**Figure 5:**
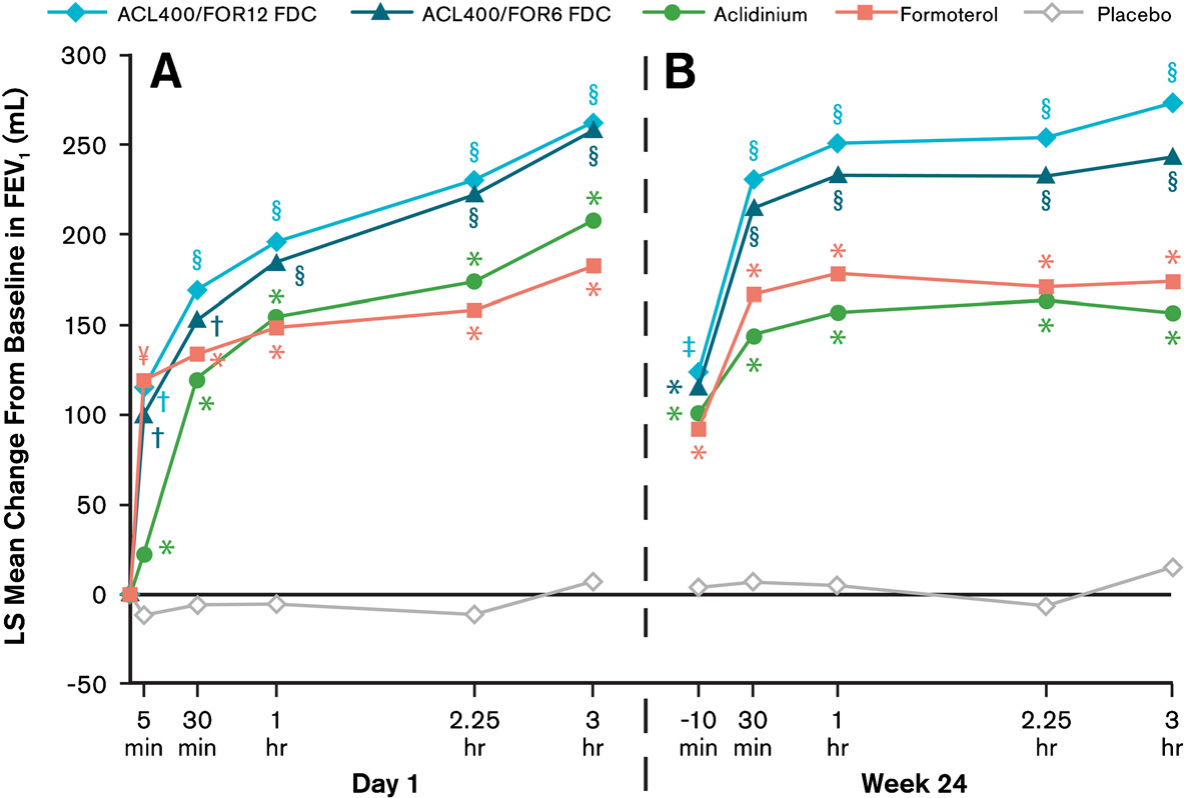
**Mean changes from baseline in FEV**_**1**_ **0–3 hours (A) on day 1 and (B) at week 24.** Analyses were based on a mixed-model for repeated measures. ^*^p < 0.05 versus placebo; ^†^p < 0.05 versus aclidinium and placebo; ^§^p < 0.05 versus aclidinium, formoterol, and placebo; ^¥^p < 0.05 versus aclidinium/formoterol FDC 400/6 and placebo. No significant differences between the two FDCs at any timepoint. ACL400/FOR12 FDC, fixed-dose combination of aclidinium 400 μg and formoterol 12 μg; ACL400/FOR6 FDC, fixed-dose combination of aclidinium 400 μg and formoterol 6 μg; FEV_1_, forced expiratory volume in 1 second; LS, least squares.

Treatment with either aclidinium/formoterol FDC resulted in rapid bronchodilation, with significant improvements in FEV_1_ over aclidinium and placebo observed within 5 minutes of the morning dose on day 1 (Figure 5A; both p < 0.0001). At week 24, FEV_1_ results over the first 3 hours postdose were similar to those observed on day 1 (Figure 5B).

A significantly greater percentage of patients in the aclidinium/formoterol FDC 400/12 μg (26.3%), aclidinium/formoterol FDC 400/6 μg (23.1%), and formoterol (28.3%) groups achieved onset of action—defined as >15% increase from baseline in FEV_1_—at 5 minutes postdose on day 1 than patients treated with aclidinium (6.5%; p < 0.0001 for both FDCs versus aclidinium, based on odds ratios) or placebo (2.1%; p < 0.01 all active treatment).

#### Peak FEV_1_

Compared to monotherapy, treatment with either aclidinium/formoterol FDC resulted in sustained improvements in lung function over the 24-week study, with significant changes from baseline in peak FEV_1_ at day 1 and week 24 (p < 0.0001 all comparisons). Compared to placebo, significant improvements in peak FEV_1_ from baseline were observed with both aclidinium/formoterol FDCs at day 1 and week 24, with LS mean treatment differences of 216 mL and 208 mL (day 1; aclidinium/formoterol FDC 400/12 μg and aclidinium/formoterol FDC 400/6 μg, p < 0.0001) and 285 mL and 259 mL (week 24; p < 0.0001 all comparisons). Changes from baseline in peak FEV_1_ with aclidinium/formoterol FDC 400/12 μg were numerically greater than those with aclidinium/formoterol FDC 400/6 μg at day 1 and week 24.

Maximal bronchodilation over placebo was achieved at 3 hours postdose on day 1 with aclidinium/formoterol FDC 400/12 μg and aclidinium/formoterol FDC 400/6 μg (258 and 255 mL, respectively), similar to the values observed at week 24 (298 and 264 mL; all p < 0.0001 versus placebo). Significantly greater improvements in peak FEV_1_ were observed on day 1 and week 24 in patients treated with either monotherapy compared with placebo (LS mean difference for aclidinium and formoterol: day 1, 165 mL and 154 mL; week 24, 174 mL and 182 mL; p < 0.0001 all comparisons).

#### 12-hour serial spirometry substudy

In a subset of ITT patients (N = 270) who participated in a 12-hour serial spirometry substudy, statistically significant changes from baseline in FEV_1_ over placebo were observed at most timepoints for those treated with either aclidinium/formoterol FDC; results for both aclidinium/formoterol FDCs were numerically greater than the monotherapies (Additional file 1: Figure S1). Data from the serial spirometry substudy support the results observed for the entire ITT population and substantiates the BID dosing regimen of the aclidinium/formoterol FDCs, as demonstrated by the FEV_1_ values over the entire dosing interval (Additional file 1: Figure S1).

#### Breathlessness

**Figure 6:**
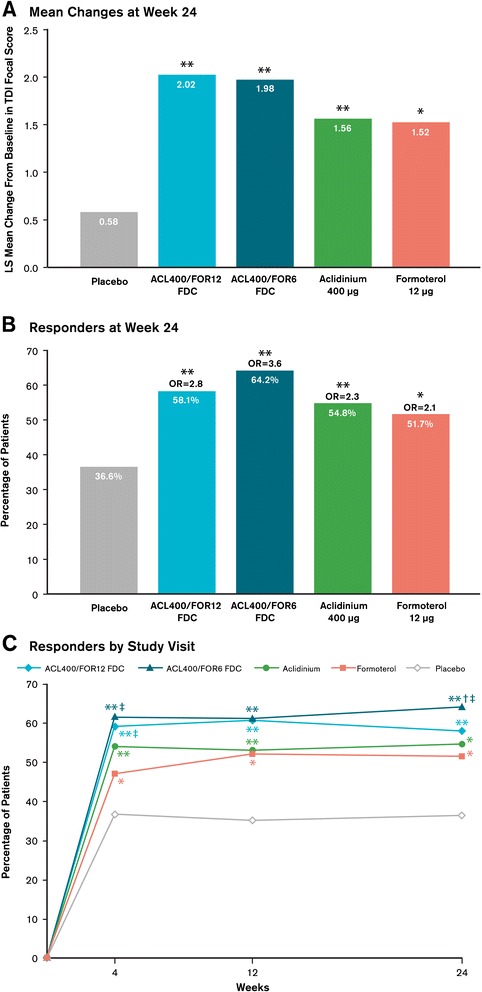
**Improvements in TDI focal score as assessed by (A) mean changes from baseline at week 24, (B) percentage of patients who responded at week 24, and (C) percentage of patients who responded over time.** Mean changes were analyzed using a mixed-model for repeated measures. Responders were defined as patients who had a TDI focal score improvements of ≥1 unit; ORs based on a logistic random effect model for active treatment versus placebo. ^*^p < 0.01 versus placebo; ^**^p ≤ 0.001 versus placebo; ^†^p < 0.05 versus aclidinium and placebo; ^‡^p < 0.05 versus formoterol and placebo. ACL400/FOR12 FDC, fixed-dose combination of aclidinium 400 μg and formoterol 12 μg; ACL400/FOR6 FDC, fixed-dose combination of aclidinium 400 μg and formoterol 6 μg; LS, least squares; OR, odds ratio; TDI, Transition Dyspnea Index.

At week 24, significant improvements in TDI focal scores were achieved with the aclidinium/formoterol FDCs compared with placebo (secondary endpoint; p < 0.0001), as well as with either aclidinium or formoterol (p ≤ 0.01 for both versus placebo; Figure 6A). Treatment with the aclidinium/formoterol FDCs resulted in numerically greater improvements in TDI focal scores compared to either monotherapy. At all other study visits, significantly greater improvements in TDI focal scores were observed with aclidinium/formoterol FDC 400/12 μg versus formoterol (p < 0.01); improvements in TDI focal scores were similar between aclidinium/formoterol FDC 400/12 μg and aclidinium/formoterol FDC 400/6 μg.

At week 24, all active treatments reached the MCID of 1-unit improvement from baseline. The percentage of patients who reached the MCID of ≥1-unit improvement from baseline in TDI focal score was greater in either aclidinium/formoterol FDC group versus placebo (p < 0.0001 based on odds ratios). Treatment with either monotherapy also resulted in significantly greater percentages of patients who achieved the MCID versus placebo at week 24 (p < 0.01), though greater odds ratios were observed with the FDCs compared to either monotherapy (Figure 6B). At week 24, average increases in TDI focal scores beyond the MCID of 1-unit improvement over placebo (considered a more stringent criteria than improvements over baseline) were observed in patients treated with either aclidinium/formoterol FDC (p < 0.0001). Both monotherapies neared the 1-unit improvement over placebo at week 24, with changes from baseline of 0.98 and 0.94 for aclidinium and formoterol, respectively. All active treatment arms resulted in significantly greater percentages of responders versus placebo throughout the study period (Figure 6C).

#### Health status

**Figure 7:**
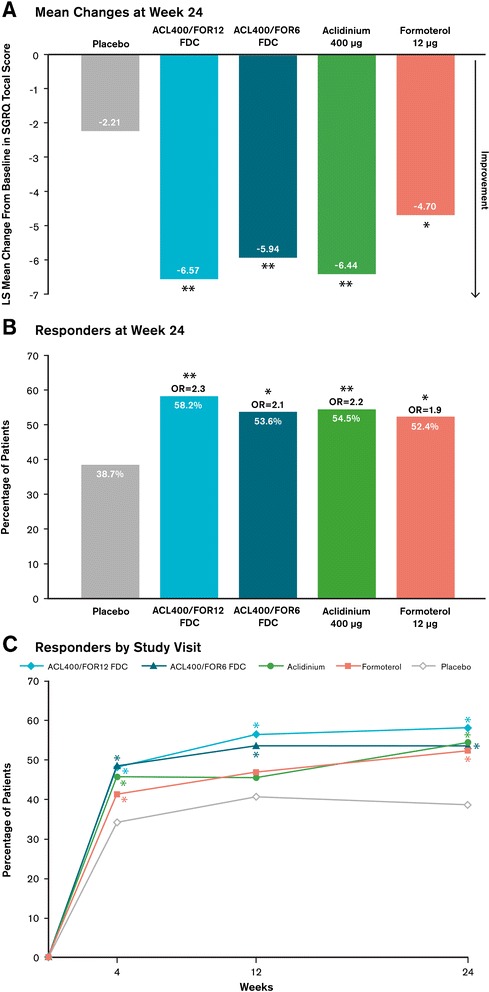
**Improvements in SGRQ total score as assessed by (A) mean changes from baseline at week 24, (B) percentage of patients who responded at week 24, and (C) percentage of patients who responded over time.** Mean changes were analyzed using a mixed-model for repeated measures. Responders were defined as patients who had a ≥4-point improvement from baseline in SGRQ total score, with ORs based on a logistic random effect model for active treatment versus placebo. ^*^p < 0.05 versus placebo; ^**^p ≤ 0.001 versus placebo. ACL400/FOR12 FDC, fixed-dose combination of aclidinium 400 μg and formoterol 12 μg; ACL400/FOR6 FDC, fixed-dose combination of aclidinium 400 μg and formoterol 6 μg; LS, least squares; OR, odds ratio; SGRQ, St. George’s Respiratory Questionnaire.

At week 24, significant improvements in SGRQ total scores from baseline were observed with the aclidinium/formoterol FDCs and the monotherapies versus placebo (secondary endpoint, p < 0.05; Figure 7A). At all timepoints, a significantly greater percentage of responders (patients achieving ≥4-unit improvement from baseline in SGRQ total score) were observed with either aclidinium/formoterol FDC versus placebo, including at study end (Figure 7B, both p < 0.01).

At week 24, mean differences in SGRQ total score over placebo (again, a more rigorous criteria than over baseline) exceeded the MCID of 4 units in patients treated with aclidinium/formoterol FDC 400/12 μg or aclidinium monotherapy (week 24; p < 0.001). Compared with placebo, treatment with either aclidinium or formoterol resulted in a significantly greater percentage of responders at weeks 4 and 24, while treatment with either aclidinium/formoterol FDC resulted in significantly greater percentages of responders at all study weeks (Figure 7C; all comparisons p < 0.05 versus placebo).

#### Rescue medication use

Compared with placebo, significant reductions in the change from baseline in overall total daily rescue medication use over 24 weeks were observed in each active treatment group (p < 0.0001). A numerically greater magnitude of effect was observed in patients treated with the aclidinium/formoterol FDCs compared to either monotherapy (−1.11 and −1.10 puffs per day for aclidinium/formoterol FDC 400/12 μg and 400/6 μg versus −0.68 aclidinium and −0.90 formoterol). Improvements from baseline in overall total daily use of rescue medication were significantly greater for both aclidinium/formoterol FDCs versus aclidinium alone (p < 0.01).

#### EXACT-RS

**Figure 8:**
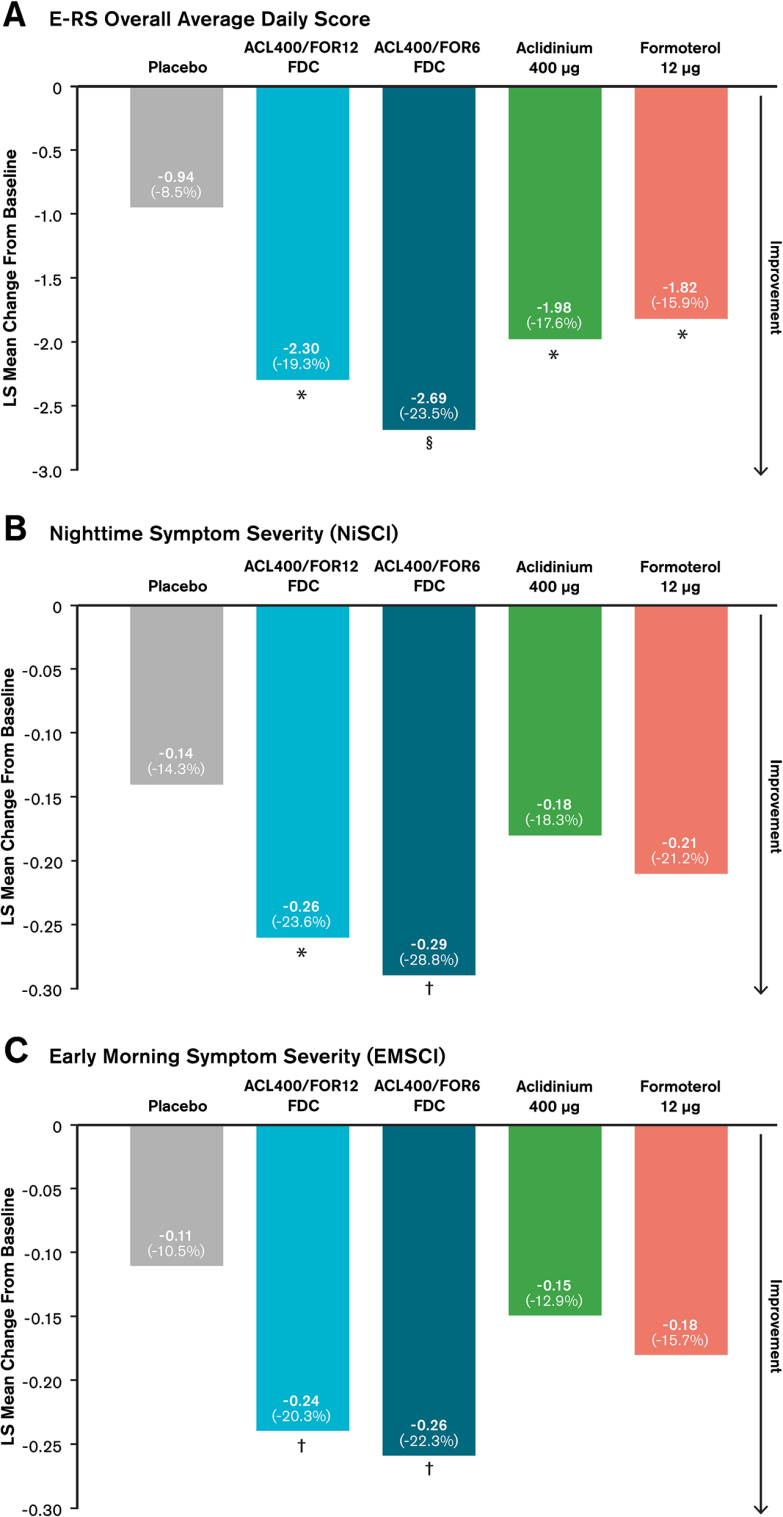
**Mean changes from baseline to week 24 in (A) E-RS overall average daily score over the study period, (B) nighttime symptom severity, and (C) early morning symptom severity.** Parenthetical values are the percent changes from baseline for the specified group. The E-RS analysis was conducted using the ITT exacerbation population, defined as all randomized patients who received ≥1 dose of double-blind study drug; the analyses of nighttime and early morning symptom severity were conducted using the general ITT population. For all outcomes, mean changes were analyzed using a mixed-model for repeated measures. ^*^p < 0.05 versus placebo; ^†^p < 0.05 versus aclidinium and placebo; ^§^p < 0.05 versus aclidinium, formoterol, and placebo. ACL400/FOR12 FDC, fixed-dose combination of aclidinium 400 μg and formoterol 12 μg; ACL400/FOR6 FDC, fixed-dose combination of aclidinium 400 μg and formoterol 6 μg; EMSCI, early morning symptoms of COPD instrument; E-RS, EXACT respiratory symptoms; ITT, intention-to-treat; LS, least square; NiSCI, nighttime symptoms of COPD instrument.

Over 24-weeks, significant improvements in overall average daily EXACT-Respiratory Symptoms scores were observed with both FDCs and the monotherapies compared with placebo (Figure 8A; p < 0.01). The changes from baseline in overall average daily E-RS scores were numerically improved for aclidinium/formoterol FDC 400/12 μg and significantly improved for aclidinium/formoterol FDC 400/6 μg versus either monotherapy (p < 0.05). For the change from baseline in E-RS total score, significant improvements from baseline were observed for all active treatment groups over placebo at all study visits (p < 0.05 for all). No consistently significant improvements were observed for either of the FDCs versus the monotherapies across visits, though both FDCs showed significant improvements compared with aclidinium and/or formoterol intermittently during the study. At all assessments, the aclidinium/formoterol FDC 400/6 μg dose resulted in numerically greater improvements than the aclidinium/formoterol FDC 400/12 μg dose.

#### Nighttime symptoms of COPD

Treatment with either aclidinium/formoterol FDC resulted in significant improvements versus placebo in overall nighttime symptom severity, measured by the NiSCI, at all study visits including study end (Figure 8B; p < 0.01 versus placebo). While treatment with either aclidinium or formoterol monotherapies significantly improved nighttime symptom severity over placebo at weeks 4 and 18 (both p < 0.05), statistical significance was not met at week 24. Patients in the aclidinium/formoterol FDC treatment groups reported greater reductions in overall nighttime COPD symptom severity versus either monotherapy at week 24, with results reaching statistical significance for the aclidinium/formoterol FDC 400/6 μg versus aclidinium (Figure 8B; p < 0.05). At all other timepoints (weeks 4, 12, and 18), both aclidinium/formoterol FDCs resulted in significant reductions in overall nighttime symptom severity versus aclidinium (p < 0.05). Significant improvements versus formoterol were observed for the aclidinium/formoterol FDC 400/12 μg dose at weeks 4 and 18, while the aclidinium/formoterol FDC 400/6 μg resulted in significant improvements in this comparison at weeks 12 and 18 (all p < 0.05). Numerically greater improvements were observed with the FDC 400/12 dose versus the FDC 400/6 dose at week 4.

#### Early morning symptoms of COPD

The average rating for overall early morning COPD symptom severity via the EMSCI was significantly improved for the aclidinium/formoterol FDCs compared with placebo at all timepoints including study end (Figure 8C; p < 0.01 for all). Neither monotherapy treatment resulted in improvements in symptom severity over placebo at any visit with the exception of formoterol at week 4 (p < 0.01). At week 24, the average rating for overall early morning COPD symptom severity via the EMSCI was significantly improved for both aclidinium/formoterol FDCs versus aclidinium (p < 0.05), but not versus formoterol. For all other timepoints, both aclidinium/formoterol FDCs significantly reduced early morning symptom severity compared with either monotherapy (p ≤ 0.01 for all), except at week 4 for aclidinium/formoterol FDC 400/12 μg versus formoterol. Improvements observed between the aclidinium/formoterol FDCs were similar throughout the study.

### Safety

The overall incidence of treatment-emergent AEs (TEAEs) with aclidinium/formoterol FDCs was similar to those of aclidinium and numerically greater than formoterol, with no apparent dose-related trends between the higher and lower FDC doses. The most commonly reported TEAEs (≥5% of patients in any treatment group) were cough and nasopharyngitis (Table 2). The majority of TEAEs were mild or moderate in severity and were considered unrelated to treatment by trial investigators. The incidences of AEs that led to discontinuation were comparable between aclidinium/formoterol FDC 400/12 μg (6.3%), aclidinium/formoterol FDC 400/6 μg (6.6%), and placebo (6.3%), and slightly lower in the aclidinium (4.7%) and formoterol (4.2%) groups. The AE most commonly associated with discontinuation was dyspnea, reported mostly in the placebo group (0.9% placebo versus ≤0.6% in active treatment arms).

**Table 2:** **Treatment-emergent adverse events** ^**a**^ **(≥2% in any treatment group)**

	**PBO (n = 332)**	**ACL400/FOR12 FDC (n = 335)**	**ACL400/FOR6 FDC (n = 333)**	**ACL400 (n = 337)**	**FOR 12 (n = 332)**
Patients with ≥1 TEAE, n (%)	181 (54.5)	215 (64.2)	203 (61.0)	210 (62.3)	189 (56.9)
TEAEs by preferred term, n (%)					
Cough^b^	12 (3.6)	17 (5.1)	13 (3.9)	7 (2.1)	10 (3.0)
Headache^b^	11 (3.3)	16 (4.8)	14 (4.2)	13 (3.9)	12 (3.6)
Nasopharyngitis	12 (3.6)	16 (4.8)	17 (5.1)	12 (3.9)	22 (6.6)
Urinary tract infection^b,c^	10 (3.0)	15 (4.5)	7 (2.1)	11 (3.3)	9 (2.7)
Back pain	9 (2.7)	10 (3.0)	5 (1.5)	4 (1.2)	6 (1.8)
Upper respiratory tract infection	5 (1.5)	10 (3.0)	13 (3.9)	11 (3.3)	9 (2.7)
Diarrhea	8 (2.4)	9 (2.7)	10 (3.0)	9 (2.7)	6 (1.8)
Muscle spasms^b^	3 (0.9)	9 (2.7)	4 (1.2)	2 (0.6)	6 (1.8)
Sinusitis	7 (2.1)	9 (2.7)	8 (2.4)	12 (3.6)	6 (1.8)
Dry mouth^c^	1 (0.3)	8 (2.4)	5 (1.5)	2 (0.6)	3 (0.9)
Tooth abscess	2 (0.6)	8 (2.4)	2 (0.6)	2 (0.6)	0
Musculoskeletal pain	2 (0.6)	7 (2.1)	0	2 (0.6)	3 (0.9)
Oropharyngeal pain	10 (3.0)	7 (2.1)	6 (1.8)	4 (1.2)	6 (1.8)
Dizziness	7 (2.1)	6 (1.8)	4 (1.2)	5 (1.5)	8 (2.4)
Insomnia^b^	2 (0.6)	6 (1.8)	3 (0.9)	3 (0.9)	9 (2.7)
Dyspnea	6 (1.8)	5 (1.5)	11 (3.3)	6 (1.8)	3 (0.9)
Nausea	4 (1.2)	5 (1.5)	15 (4.5)	12 (3.6)	14 (4.2)
Hypertension^b^	6 (1.8)	4 (1.2)	9 (2.7)	10 (3.0)	9 (2.7)
Constipation^b,c^	6 (1.8)	3 (0.9)	4 (1.2)	7 (2.1)	4 (1.2)
Pain in extremity	0	3 (0.9)	3 (0.9)	7 (2.1)	2 (0.6)
Vomiting	2 (0.6)	3 (0.9)	7 (2.1)	5 (1.5)	3 (0.9)
Fatigue	8 (2.4)	2 (0.6)	6 (1.8)	4 (1.2)	7 (2.1)
Gastroenteritis viral	8 (2.4)	2 (0.6)	5 (1.5)	3 (0.9)	2 (0.6)

^a^Per protocol exacerbations of COPD were not considered an *a priori* safety outcome and are therefore not included within the safety results; ^b^Potential β_2_-agonist TEAE; ^c^Potential anticholiergic TEAE.

ACL 400, aclidinium 400 μg; ACL400/FOR12 FDC, fixed-dose combination of aclidinium 400 μg and formoterol 12 μg; ACL400/FOR6 FDC, fixed-dose combination of aclidinium 400 μg and formoterol 6 μg; FOR 12, formoterol 12 μg; PBO, placebo; TEAE, treatment-emergent adverse event.

Of the class-related AEs that may occur due to both anticholinergic and β_2_-agonist mechanisms, only urinary tract infection occurred in >2% of patients in any treatment group (Table 2). The most common (>2% and >placebo) potential anticholinergic AEs that occurred with aclidinium/formoterol FDC 400/12 μg versus aclidinium alone were dry mouth (2.4% versus 0.6%) and oropharyngeal pain (2.1% versus 1.2%). The most common (>2% and >placebo) β_2_-agonist AEs with aclidinium/formoterol FDC 400/12 μg versus formoterol alone were cough (5.1% versus 3.0%), headache (4.8% versus 3.6%), and muscle spasm (2.7% versus 1.8%).

The overall incidence of serious AEs (SAEs) was low and numerically higher in all active treatment arms compared with placebo (5.7% aclidinium/formoterol FDC 400/12 μg, 5.4% aclidinium/formoterol FDC 400/6 μg, 5.0% aclidinium, 4.5% formoterol, and 3.6% placebo). Pneumonia, the most common SAE, was reported by no more than 3 (0.9%) patients in any randomized group (2 patients in the aclidinium/formoterol FDC 400/12 μg group, 1 in aclidinium/formoterol FDC 400/6 μg, 1 in aclidinium, 3 in formoterol, and 3 in placebo); none of the reported cases of pneumonia were considered treatment related. Overall, 3 patients experienced an SAE that was considered related to treatment (1 each in the placebo [atrial fibrillation], aclidinium/formoterol FDC 400/12 μg [pneumonitis], and formoterol groups [atrial fibrillation], with only the placebo-treated patient discontinuing due to the SAE).

The number of Major Adverse Cardiac Events (MACEs), based on blinded adjudication, TEAEs and SAEs, were infrequent and occurred at similar incidences across all treatment groups. All adjudicated MACEs were SAEs with the exception of 1 report of moderate nonfatal stroke in the placebo group. Based on adjudicated SAEs, a total of 12 MACEs were reported for 12 patients. MACEs based on adjudicated SAEs were reported in 2 (0.6%) and 4 (1.2%) patients in the aclidinium/formoterol FDC 400/12 μg and 400/6 μg treatment groups. A total of 2 (0.6%) patients in the placebo group, 1 (0.3%) patient in the aclidinium group, and 3 (0.9%) patients in the formoterol group reported MACEs. All MACEs were considered unrelated to treatment.

A total of 5 deaths occurred during the treatment period or within 30 days of the last dose of investigational product, none of which were considered related to treatment. One death occurred in the aclidinium/formoterol FDC 400/12 μg group, 3 in the aclidinium group, and 1 in the formoterol group. Three of these deaths (1 each in the aclidinium/formoterol FDC 400/12 μg, aclidinium, and formoterol groups) were adjudicated as CV deaths (etiology unknown). The other two deaths, both occurring in the aclidinium group, were due to esophageal adenocarcinoma and gastrointestinal necrosis.

Mean changes from baseline in clinical laboratory parameters, vital signs, and ECGs were small and of no clinical relevance. Holter monitoring did not show any findings of an ECG effect for patients in any group, and no differences were observed between the treatment arms.

## Discussion

An FDC comprising bronchodilators with complementary mechanisms of action may improve lung function, while offering patients the convenience of drug delivery via a single device without increasing the risk for adverse events [20]. Though the interaction between LAMAs and LABAs has not been definitively determined, LABAs have been shown to enhance the bronchodilatory effect of LAMAs through a decrease in acetylcholine transmission that leads to a reduction in bronchoconstriction, while LAMAs amplify the effect of LABAs by blocking the muscarinic receptors targeted by acetylcholine, resulting in further bronchodilation [3,21,22]. Thus, a fixed-dose combination of a LAMA and LABA is an important therapeutic option, providing patients with more convenient drug delivery and the potential for improved compliance.

In this trial, treatment with the LAMA/LABA fixed dose combination of aclidinium/formoterol FDC 400/12 μg for 24 weeks resulted in statistically significant and clinically meaningful improvements for the coprimary measures of lung function: 1-hour morning postdose FEV_1_ versus aclidinium monotherapy (contribution of formoterol) and morning trough FEV_1_ versus formoterol (contribution of aclidinium).

The coprimary endpoints in the AUGMENT COPD study reported here reflect FDA guidance regarding fixed-dose combination drugs (ie, each component of the fixed-dose combination must make a contribution to the claimed effects [23]). One aspect behind the rationale for combining aclidinium and formoterol for this FDC was the difference in time course for effective bronchodilation between the two drugs. Formoterol was expected to provide a rapid onset of action while the contribution of aclidinium was expected to occur over many hours (at trough) [11,24]. The trial was thus powered to detect differences in the prespecified coprimary endpoint comparisons: 1-hour morning postdose FEV_1_ for the FDCs versus aclidinium (to observe the early contribution of formoterol) and trough FEV_1_ for the FDCs versus formoterol (to observe the contribution of aclidinium over many hours). Any other comparisons for these outcomes were considered supportive in nature.

At study end, the aclidinium/formoterol 400/12 μg FDC provided an additional 45 mL in trough FEV_1_ from baseline (contribution of aclidinium), while the contribution of formoterol to the FDC (aclidinium/formoterol FDC 400/12 μg versus aclidinium) was 28 mL, indicating that aclidinium provided greater bronchodilation. Results for trough FEV_1_ over the course of the study support the endpoint observation that the contribution of formoterol as a component of the FDC was smaller than that of aclidinium. Additionally, trough FEV_1_ values for both FDCs were numerically greater than aclidinium at all timepoints throughout the study, a result that is supportive of the observation that the FDCs provide greater bronchodilation than either monotherapy component alone.

Throughout the study, improvements in lung function with aclidinium/formoterol FDC 400/12 μg, which were numerically greater than those with aclidinium/formoterol FDC 400/6 μg, were generally similar to the results observed in a similarly designed study (ACLIFORM COPD) [25]. Results from this trial also demonstrate rapid bronchodilation with aclidinium/formoterol FDC treatment (within 5 minutes of dosing) that was sustained and clinically meaningful in patients with COPD. Both aclidinium/formoterol FDCs had safety profiles generally similar to that of each monotherapy, though there were a numerically greater percentage of FDC- and aclidinium-treated patients who experienced any AE compared with those treated with formoterol. The incidence of MACE was comparable among all active treatment groups. Together, these results indicate that treatment with a fixed-dose combination of aclidinium/formoterol achieves a level of bronchodilation greater than either monotherapy component and is well-tolerated in patients with moderate to severe COPD. The therapeutic benefits on lung function derived from treatment with the aclidinium/formoterol FDCs exceeded the recommended MCID of 100 mL in 1-hour morning postdose FEV_1_ versus placebo and were consistently significantly greater than those of the monotherapies. These improvements were observed from the first timepoint assessed until the end of the study, demonstrating sustained bronchodilation throughout 24 weeks of treatment.

Onset of bronchodilation (>15% increase from baseline in FEV_1_) with aclidinium/formoterol FDC 400/12 μg was observed as early as 5 minutes after the first dose, similar to that of formoterol—a LABA known to have a fast onset of action [26]. The improved efficacy with the aclidinium/formoterol LAMA/LABA combination over the monotherapy components and placebo may be attributed to complementary pharmacodynamic profiles of these 2 bronchodilators: direct bronchodilation by the β_2_-agonist, formoterol, provides rapid onset of action, while reduction in bronchoconstriction by the antimuscarinic, aclidinium, prolongs duration of bronchodilation [24,27]. As rapid onset of effect has been associated with better patient compliance [28,29], the onset of action observed with the aclidinium/formoterol FDCs may have a positive effect on medication adherence in clinical practice.

Although spirometric outcomes are important in assessing airflow obstruction in patients with COPD, clinical measures of health status have been shown to correlate better with symptoms such as breathlessness [30,31]—one of the most troublesome symptoms of the disease that often contributes to limitations in patients’ activities [30-32]. Following 24 weeks of treatment, improvements in SGRQ total score exceeded the MCID over placebo in patients treated with either aclidinium/formoterol FDC 400/12 μg or aclidinium monotherapy. Patients treated with aclidinium/formoterol FDC 400/12 μg also experienced improvements in TDI focal score that exceeded the MCID over placebo. Compared with placebo, a significantly greater percentage of aclidinium/formoterol FDC 400/12 μg and 400/6 μg-treated patients reached the MCID for both SGRQ and TDI at all study visits. These results support the clinical benefit of aclidinium/formoterol FDCs in improving health status and in reducing breathlessness, important treatment goals for the effective management of COPD [32].

A recently published review of the applicability of MCIDs in COPD trials outlines numerous challenges when comparing combination therapies to monotherapy [33]. The authors indicate that improvements in various outcome measures with combination therapy over monotherapy should not be expected to exceed those of monotherapy over placebo or to produce a result that would reach an MCID. Further, the authors suggest that MCIDs or responder rates derived from trials comparing a single active agent to placebo may not be applicable to combination therapy trials in which the comparison is to each monotherapy component. It is not surprising that the observed differences between monotherapy and placebo are often greater than the differences between combination therapy and the monotherapy components [34]. To describe the additional proportion of patients who may experience improvements at or above the MCID following the addition of one active treatment to another, the concept of a “minimum worthwhile incremental advantage” has been proposed [33]. In light of the caveats inherent in combination versus monotherapy trials, as well as the recognition that patients may experience advantages with combination therapy that are not readily measurable by certain outcome criteria, it is reasonable to conclude that the improvements in lung function and symptoms observed with the aclidinium/formoterol FDCs over each monotherapy in this trial may have clinical benefits for the moderate to severe COPD patient.

Due to the circadian nature of cholinergic tone, more impaired lung function is observed in the evening versus daytime in patients with COPD [35]. This in turn may be related to the prevalence of sleep disturbance in a majority of patients with COPD [36,37], as well as reports of nighttime and early morning being the worst times of day for COPD patients due to breathlessness and other symptoms [38]. Twice-daily aclidinium has been shown to significantly improve lung function at night compared with once-daily tiotropium [8], while other twice-daily COPD medications, including formoterol, reportedly improve nighttime symptoms [39-42]. In the trial reported here, aclidinium/formoterol FDCs significantly reduced both nighttime and early morning symptoms compared with placebo—measured by the newly developed NiSCI and EMSCI patient reported outcome measures—while treatment with the monotherapy components generally did not reach statistical significance in these outcomes. The disparity between nighttime symptoms outcomes with aclidinium monotherapy in this trial with those in a previously conducted study [43] could be due to the manner in which nighttime symptoms were evaluated as both the NiSCI and the EMSCI are currently undergoing empirical testing.

As the current study demonstrated that aclidinium/formoterol administered in the morning significantly improves bronchodilation as rapidly as 5 minutes postdose, the evening dose of this twice-daily treatment may also alleviate impaired airflow at night and reduce breathlessness, potentially providing the added benefit of improving COPD symptoms when they are at their worst. Further analyses are necessary to correlate the clinically meaningful treatment effect of the aclidinium/formoterol FDCs on lung function with the positive effects on COPD symptoms.

## Conclusions

The spirometric and clinical outcomes from this study demonstrate the sustained and improved efficacy of a fixed-dose combination of aclidinium 400 μg/formoterol 12 μg over its monotherapy components. With a safety profile generally similar to the aclidinium and formoterol monotherapies, the results reported here support the use of an aclidinium/formoterol FDC as maintenance treatment for patients with moderate to severe COPD.

## Additional file

Additional file 1: Table S1. Schedule of Evaluations. **Figure S1.** Change from baseline in FEV_1_ by timepoint at week 24. This analysis was conducted in a subset of patients from the ITT population who participated in the 12-hour serial spirometry substudy. ACL400/FOR12 FDC, fixed-dose combination of aclidinium 400 μg and formoterol 12 μg; ACL400/FOR6 FDC, fixed-dose combination of aclidinium 400 μg and formoterol 6 μg; FEV_1_, forced expiratory volume in 1 second; ITT, intention-to-treat; LS, least square.

## Abbreviations

AE: Adverse event
ACL: Aclidinium
BDI: Baseline dyspnea index
BID: Twice daily
COPD: Chronic obstructive pulmonary disease
CV: Cardiovascular
ECG: Electrocardiogram
EMSCI: Early morning symptoms of COPD instrument
EXACT-RS: EXAcerbations of Chronic pulmonary disease Tool – Respiratory Symptoms
FDC: Fixed-dose combination
FEV_1_: Forced expiratory volume in 1 second
FOR: Formoterol
FVC: Forced vital capacity
ICS: Inhaled corticosteroids
ITT: Intention-to-treat
LABA: Long-acting β_2_-agonist
LAMA: Long-acting muscarinic antagonist
LS: Least square
MACE: Major adverse cardiovascular events
MCID: Minimal clinically important difference
MedDRA: Medical dictionary for regulatory activities
MI: Myocardial infarction
MMRM: Mixed model for repeated measures
NiSCI: Nighttime Symptoms of COPD Instrument
SAE: Serious adverse event
SGRQ: St. George’s respiratory questionnaire
TDI: Transition dyspnea index
