# Supplementary material for: Efficacy and safety of fixed-dose combinations of aclidinium bromide/formoterol fumarate: the 24-week, randomized, placebo-controlled AUGMENT COPD study
Source: Respir Res. 2014 Oct 14;15(1):123. doi: 10.1186/s12931-014-0123-0 (PMC4213545; doi:10.1186/s12931-014-0123-0)

Supplemental Data

Table. Schedule of evaluations

Evaluation	Run-in	Double-Blind Treatment						
	**Visit 1 Screening**	**Visit 2 Day 1**	**Visit 3 Day 4**	**Visit 4 Week 4**	**Visit 5 Week 12**	**Visit 6 Week 18**	**Visit 7 Week 24**	**ET Visit**
Predose PFT[a]		X	X	X	X	X	X	X
Postdose PFT[b]		X	X	X	X		X	X
BDI/TDI[a]		X		X	X		X	
SGRQ		X		X	X		X	X
EXACT[c]	X	X	X	X	X	X	X	X
EMSCI[c]	X	X	X	X	X	X	X	X
NiSCI[c]	X	X	X	X	X	X	X	X
12-hour PFT (spirometry substudy)[d]		X			X		X	
Rescue medication use[c]	X	X	X	X	X	X	X	X
Adverse events	X	X	X	X	X	X	X	X
Vital signs[c]	X	X	X	X	X	X	X	X
12-lead ECG[d]	X	X	X	X	X		X	X
Laboratory tests (fasting)	X				X		X	X

[a]IC conducted before forced maneuvers. FEV_1 and FVC performed at 1 hour and 10 minutes predose except at ET (only once if patient was able to complete the tests).

[b]FEV_1 and FVC performed at 30 minutes, 1 hour, and 2.25 hours postdose. At Visit 2, an additional evaluation was taken at 5 minutes post-dose.

[a]BDI/TDI was always completed before the SGRQ. BDI was performed at Visit 2 (predose); TDI was performed at subsequent visits.

[b]Recorded twice-daily (morning and evening) in the patient electronic diary.

[c]Blood pressure and heart rate readings taken after patient had been sitting for 10 minutes. Body weight was evaluated at Visit 1 and Visit 7/ET.

[d]Performed in approximately 20% of patients at selected sites. FEV_1 and FVC evaluated at 4, 6, 8, 10, and 12 hours post- morning dose. IC only performed at 12 hours post-morning dose.

[d]ECG performed pre-dose and 2 hours post-dose except at Visit 1 (once for eligibility only) and at ET Visit (once).

BDI, Baseline Dyspnea Index; ECG, electrocardiogram; ET, early termination; EMSCI, Early Morning Symptoms of COPD Instrument; EXACT, Exacerbation of Chronic Pulmonary Disease Tool; FEV, forced expiratory volume in 1 second; FVC, forced vital capacity; NiSCI, Nighttime Symptoms of COPD Instrument; PFT, pulmonary function test; SGRQ, St. George's Respiratory Questionnaire; TDI, Transition Dyspnea Index.

Figure. Change from baseline in FEV_1 by timepoint at week 24. This analysis was conducted in a subset of patients from the ITT population who participated in the 12-hour serial spirometry substudy. ACL400/FOR12 FDC, fixed-dose combination of aclidinium 400 µg and formoterol 12 µg; ACL400/FOR6 FDC, fixed-dose combination of 400 µg and formoterol 6 µg; FEV_1, forced expiratory volume in 1 second; ITT, intention-to-treat; LS, least square

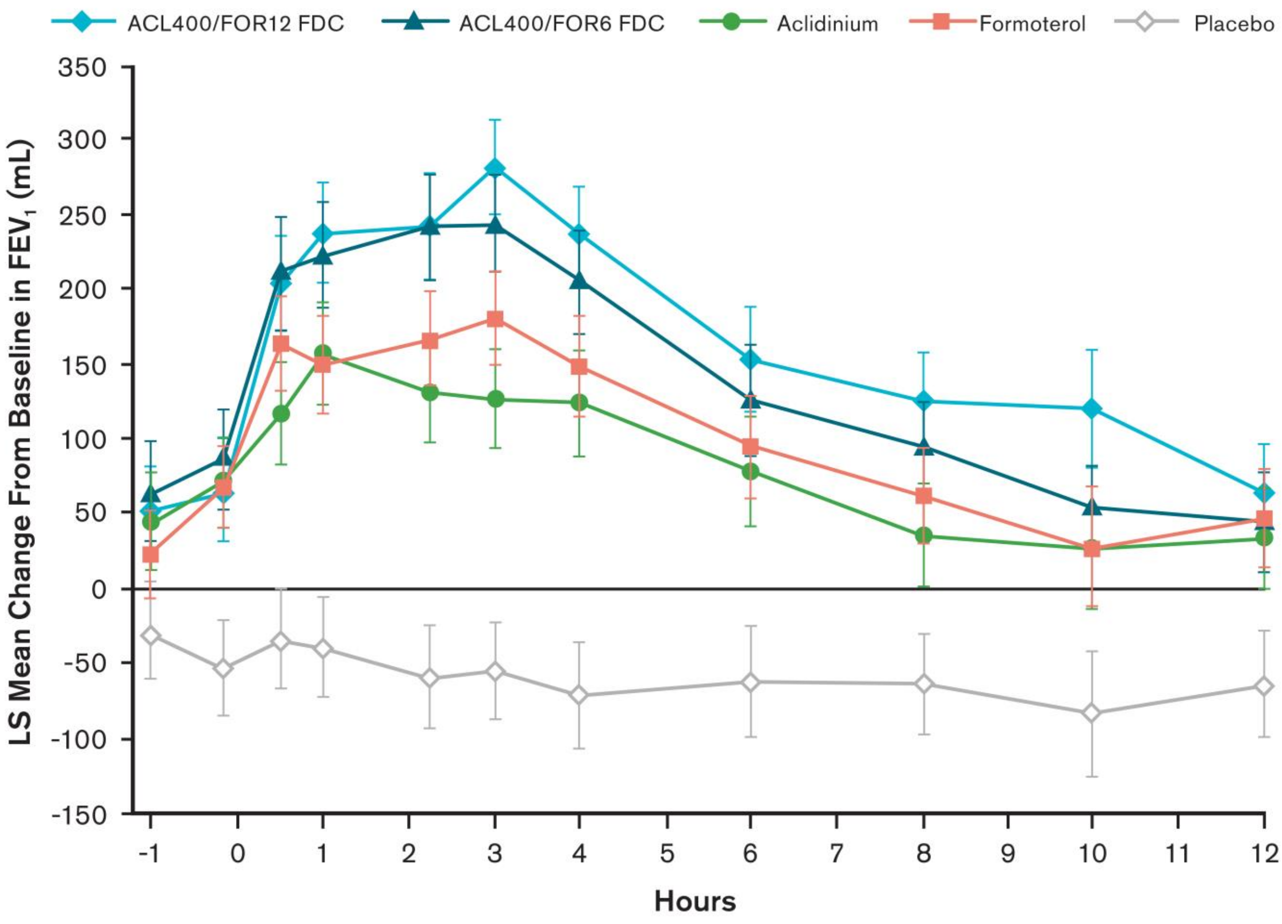

Supplement: Additional file 1: Table S1. — Schedule of Evaluations. Figure S1. Change from baseline in FEV1 by timepoint at week 24. This analysis was conducted in a subset of patients from the ITT population who participated in the 12-hour serial spirometry substudy. ACL400/FOR12 FDC, fixed-dose combination of aclidinium 400 μg and formoterol 12 μg; ACL400/FOR6 FDC, fixed-dose combination of aclidinium 400 μg and formoterol 6 μg; FEV1, forced expiratory volume in 1 second; ITT, intention-to-treat; LS, least square. [file 12931_2014_123_MOESM1_ESM.pdf]
